# Global excess deaths associated with heatwaves in 2023 and the contribution of human-induced climate change

**DOI:** 10.1016/j.xinn.2025.101110

**Published:** 2025-09-04

**Authors:** Samuel Hundessa, Wenzhong Huang, Rongbin Xu, Zhengyu Yang, Qi Zhao, Antonio Gasparrini, Ben Armstrong, Michelle L. Bell, Veronika Huber, Aleš Urban, Micheline Coelho, Francesco Sera, Shilu Tong, Dominic Royé, Jan Kyselý, Francesca de'Donato, Malcolm Mistry, Aurelio Tobias, Carmen Íñiguez, Martina S. Ragettli, Simon Hales, Souzana Achilleos, Jochem Klompmaker, Shanshan Li, Yuming Guo, Multi-Country Multi-City Collaborative Research Network

**Affiliations:** 1 Climate, Air Quality Research Unit, School of Public Health and Preventive Medicine, Monash University, Melbourne, VIC, Australia; 2 Department of Epidemiology, School of Public Health, Cheeloo College of Medicine, Shandong University, Jinan, China; 3 Environment & Health Modelling (EHM) Lab, Department of Public Health Environments and Society, London School of Hygiene & Tropical Medicine, London, UK; 4 Department of Public Health Environments and Society, London School of Hygiene & Tropical Medicine, London, UK; 5 School of the Environment, Yale University, New Haven, CT, USA; 6 Doñana Biological Station (EBD), Spanish National Research Council (CSIC), Sevilla, Spain; 7 Institute of Epidemiology, Helmholtz Zentrum München – German Research Center for Environmental Health (GmbH), Neuherberg, Germany; 8 Institute of Atmospheric Physics of the Czech Academy of Sciences, Prague, Czech Republic; 9 Faculty of Environmental Sciences, Czech University of Life Sciences, Prague, Czech Republic; 10 Department of Pathology, Faculty of Medicine, University of São Paulo, São Paulo, Brazil; 11 Department of Statistics, Computer Science and Applications “G. Parenti,” University of Florence, Florence, Italy; 12 National Institute of Environmental Health, Chinese Center for Disease Control and Prevention, Beijing, China; 13 School of Public Health and Social Work, Queensland University of Technology, Brisbane, QLD, Australia; 14 Biological Mision of Galicia (MBG) – Spanish Council for Scientific Research (CSIC), Spain; 15 Climate Research Foundation (FIC), Madrid, Spain; 16 Spanish Consortium for Biomedical Research in Epidemiology and Public Health (CIBER Epidemiology and Public Health-CIBERESP), Madrid, Spain; 17 Department of Epidemiology, Lazio Regional Health Service, Rome, Italy; 18 Department of Economics, Ca’ Foscari University of Venice, Venice, Italy; 19 Institute of Environmental Assessment and Water Research (IDAEA), Spanish Council for Scientific Research (CSIC), Barcelona, Spain; 20 School of Tropical Medicine and Global Health, Nagasaki University, Japan; 21 Department of Statistics and Computational Research. Universitat de València, València, Spain; 22 Swiss Tropical and Public Health Institute, Allschwill, Switzerland; 23 University of Basel, Basel, Switzerland; 24 Department of Public Health, University of Otago, Wellington, New Zealand; 25 Department of Primary Care and Population Health, University of Nicosia Medical School, Nicosia, Cyprus; 26 RIVM National Institute for Public Health and the Environment, Bilthoven, Utrecht, the Netherlands

**Keywords:** all-cause mortality, heatwaves, global burden of disease, excess death, death rate, human-induced climate change

## Abstract

An unprecedented heatwave swept the globe in 2023, marking it one of the hottest years on record and raising concerns about its health impacts. However, a comprehensive assessment of the heatwave-related mortality and its attribution to human-induced climate change remains lacking. We aim to address this gap by analyzing high-resolution climate and mortality data from 2,013 locations across 67 countries/territories using a three-stage modeling approach. First, we estimated historical heatwave-mortality associations using a quasi-Poisson regression model with distributed lag structures, considering lag effects, seasonality, and within-week variations. Second, we pooled the estimates in meta-regression, accounting for spatial heterogeneity and potential changes in heatwave-mortality associations over time. Third, we predicted grid-specific (0.5° × 0.5°) association in 2023 and calculated the heatwave-related excess deaths, death ratio, and death rate per million people. Attribution analysis was conducted by comparing heatwave-related mortality under factual and counterfactual climate scenarios. We estimated 178,486 excess deaths (95% empirical confidence interval [eCI], 159,892−204,147) related to the 2023 heatwave, accounting for 0.73% of global deaths, corresponding to 23 deaths per million people. The highest mortality rates occurred in Southern (120, 95% eCI, 116−126), Eastern (107, 95% eCI, 100−114), and Western Europe (66, 95% eCI, 62**−**70), where the excess death ratio was also higher. Notably, 54.29% (95% eCI, 45.71%**−**61.36%) of the global heatwave-related deaths were attributable to human-induced climate change. These results underscore the urgent need for adaptive public health interventions and climate mitigation strategies to reduce future mortality burdens in the context of increasing global warming.

## Public summary

• Over 178,000 global deaths associated with the 2023 heatwave, equivalent to 23 deaths per million.
• Southern and Eastern Europe had the highest heatwave-related mortality burden.
• Heatwave-related deaths were concentrated in subtropical and temperate zones of the Northern Hemisphere.
• More than half (54.29%) of heatwave-related deaths were attributable to human-induced climate change.

## Graphical abstract

**Figure:**
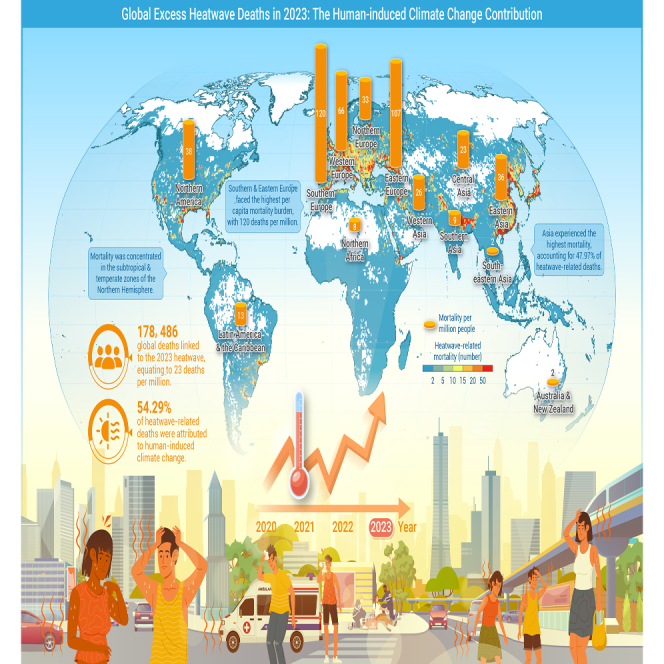

## Introduction

Anthropogenic greenhouse gas emissions have accelerated climate change, with the global average temperature between 2011 and 2020 reaching 1.1°C above the pre-industrial level.^1^^,^^2^^,^^3^ The last 9 years have been the warmest on record, culminating in 2023 being the hottest year since the beginning of global temperature monitoring.^2^^,^^3^^,^^4^ During that year, the average temperature rose to 1.45°C above pre-industrial levels.^2^^,^^3^ Every month from June to December 2023 experienced higher temperatures than the same months in any preceding year,^3^ with forecasts suggesting even higher temperatures ahead.^1^ As a result, heatwaves have become more frequent, intense,^5^ and widespread, with several countries experiencing record-high temperatures.^1^^,^^6^ Without strong mitigation and adaptation efforts, heatwave exposure and its public health impacts will worsen.^7^ Heatwaves substantially affect human health, increasing the risk of illness and death.^8^^,^^9^ From 1998 to 2017, over 166,000 heatwave-related deaths occurred worldwide, including about 70,000 in Europe in 2003^9^ and around 55,000 in Russian in 2010.^10^ Global population exposure to heatwaves rose by about 125 million between 2000 and 2016.^11^ Beyond direct impacts such as heatstroke, heatwaves exacerbate existing chronic conditions such as cardiovascular, respiratory, kidney diseases, diabetes, and mental health issues,^12^ thus escalating public health threat.

Several studies have reported a significant association between heatwaves and increased mortality risk.^8^^,^^13^^,^^14^ However, these studies have been geographically restricted and used diverse modeling techniques that hinder direct comparisons and the generalizability of the results. Efforts to estimate the burden of heatwave-related mortality have also been hampered by the limited availability of observed mortality data and methodological challenges. Some large-scale studies identified a widespread link between heatwaves and mortality across various regions,^8^^,^^15^ but they have not specifically quantified global burden of heatwave-related mortality. One recent study, for example, estimated that heatwaves have caused over 150,000 deaths per warm season globally during 1990–2019.^16^ However, it neither clearly defines “hot months” nor assesses the role of climate change. Other studies evaluating the impact of rising global temperatures have used differing heat metrics,^17^ and the mortality impact of recent heatwaves, such as the 2023 heatwave, remains unknown. Furthermore, there is a lack of comprehensive evidence on the contribution of human-induced climate change to heatwave-related mortality.

## Materials and methods

### Data sources

**Figure 1:**
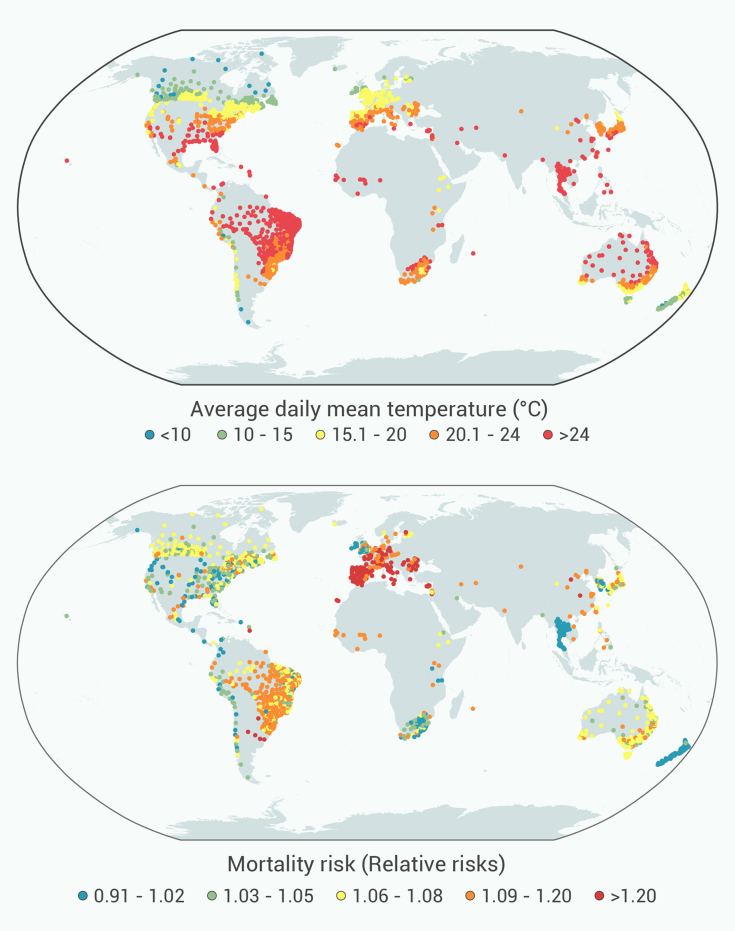
Geographical distribution of temperature and heatwave-mortality risk (A) Average daily mean temperature (°C) and locations included in the study across the world during 1973–2019. (B) The relative risk (RR) of mortality associated with heatwaves. This represents the best linear unbiased predictions obtained from the random-effect meta-analytical model applied to the whole historical data (1973–2019)—the difference in the RR indicates regional differences in heatwave impacts.

We obtained daily mortality data for each location from the Multi-Country Multi-City (MCC) Collaborative Research Network database.^7^^,^^18^^,^^19^ Additional mortality data were collected from the Cause of Death Unit Record File by Statistical Area Level 3 for Australia, the mortality data source of the New Zealand health authority, for Brazil Mortality Information System, and the health and demographic surveillance systems sites within the International Network for the Demographic Evaluation of Populations and their Health (INDEPTH) Network database.^20^ These datasets were then combined to produce a unified database for the study. To avoid duplication, MCC locations in Australia, Brazil, Canada, Chile, and Taiwan were excluded. Finally, we included mortality data from 2,013 locations in 67 countries/territories (Table S1). Mortality was represented by all-cause or otherwise by non-external deaths defined by ICD-10 codes A00-R99 or ICD-9 codes 0-799. We used the daily mean temperature as a metric for exposure. Figure 1 presents the geographic distribution of daily mean temperatures across the study locations.

We additionally collected a number of meta-predictors that were strongly associated with temperature-related deaths from more than 200 countries/territories worldwide to account for potential heterogeneity across locations. Grid-level mean temperature was accessed from the Fifth Generation of European Reanalysis (ERA5) dataset.^21^ We obtained hourly gridded temperature at a spatial resolution of 0.25° × 0.25°, which was then used to calculate daily meteorological parameters according to the local time zone of each grid cell. These grids were grouped into 0.5° × 0.5° cells by averaging values of all the 0.25° × 0.25° grids within each 0.5° × 0.5° grid cell. We also included other meta-variables, such as indicators of climatic zones (Köppen-Geiger climate classification system),^22^ gross domestic product (GDP) per capita derived from country-specific annual GDP, and population per grid cell (0.5° × 0.5°) accessed from the Global Carbon Project.^19^ The mortality rates for the period of 2000–2019 were obtained from the World Bank. All variables were assessed for missing values before analysis.

To assess the heatwave-related deaths attributable to human-induced climate change, we collected daily mean temperature simulated by four climate models from the globally gridded climate database (http://damip.lbl.gov/) for 2023 under two climate change scenarios: one reflecting actual climate conditions (factual) and the other simulating a hypothetical climate without anthropogenic influence (counterfactual) (Table S2). We bias-corrected the climate model data,^17^ to align it with the ERA5 temperature data that we used for estimating heatwave-related mortality. The supplemental information contains details of the climate data used for this study.

### Heatwave definition

Currently, there is no universally accepted definition for a heatwave, and different heatwave definitions have been used in previous studies.^15^^,^^23^^,^^24^ The studies used different temperature metrics, such as minimum, maximum, or mean temperatures.^8^^,^^15^^,^^24^ Some studies have found a comparable association between heatwaves and mortality across different temperature metrics,^15^ while others suggest that mean temperature may provide better estimates than maximum or minimum temperatures.^25^ In this study, we defined a heatwave as two or more consecutive days with a daily mean temperature above the location-specific 95th percentile of the year-round temperature distribution during 1973–2019. A binary heatwave variable (1 = heatwave or 0 = non-heatwave) was assigned to each day and location. To estimate grid-specific mortality for the study year, we calculated heatwave days using gridded temperature data based on this definition. Heatwave days refer to days within such periods that meet specific temperature and duration criteria defining a heatwave. This approach is consistent with previous multi-country studies.^7^^,^^16^

### Statistical analysis

We estimated global and regional mortality attributable to heatwaves at a spatial resolution of 0.5° × 0.5°. We utilized location-specific historical mortality data from 1973 to 2019 to calibrate the epidemiological models, which were then used to estimate grid-specific heatwave-related mortality during the hottest months of 2023. The consecutive hottest months were selected based on historical temperature patterns and hemispheric location. Accordingly, May, June, July, and August were considered the hottest months in the Northern Hemisphere, while November, December, January, and February were identified as the hottest months in the Southern Hemisphere.

The analysis consists of three main stages: (1) assessing location-specific heatwave-mortality associations, (2) pooling location-specific effect estimates through meta-regression, and (3) prediction of grid-specific mortality risk and excess deaths.^16^^,^^19^ In the first stage, we used a quasi-Poisson time-series regression model to estimate the cumulative heatwave-mortality association per location and 5-year period. Heatwave effects were modeled using a distributed lag model with a natural cubic spline (4 degrees of freedom) over a 10-day lag, capturing the delayed effects of heatwave on mortality following previous multi-country studies.^7^^,^^16^^,^^18^^,^^24^ We controlled for seasonality and long-term trend using categorical variables representing year and calendar month (e.g., 2019-Jan, 2019-Feb), and adjusted for within-week variation using day-of-week indicators.^7^^,^^24^ To consider for potential long-term changes in the heatwave-mortality association, we conducted a time-stratified analysis, dividing the epidemiological data into consecutive 5-year periods (e.g., 1973−1977, 1978−1982, etc.). Separate models were fitted to each period and location to estimate the cumulative relative risk (RR) of heatwaves on mortality.

In the second stage, we developed a random-effects meta-regression model using five location-specific meta-predictors, including climate classification, geographic region, GDP per capita, average temperature, and temperature range. These meta-predictors were chosen based on the prior evidence of their impact on the spatial heterogeneity of the heatwave-mortality association.^18^ To account for potential changes in the association over time (e.g., due to population adaptation or acclimatization), we included a time variable corresponding to the midpoint of each period as a linear fixed effect.^26^ Two interaction terms were also added to improve explanatory power: one between climate classifications and annual average temperature, and another between average temperature and temperature range. The first term addresses the varying effects of temperature across different climate zones,^27^^,^^28^ while the second captures difference in temperature variability.^29^ The model incorporating these interactions accounted for a substantially larger proportion of heterogeneity than the model without them (Table S3).

**Figure 2:**
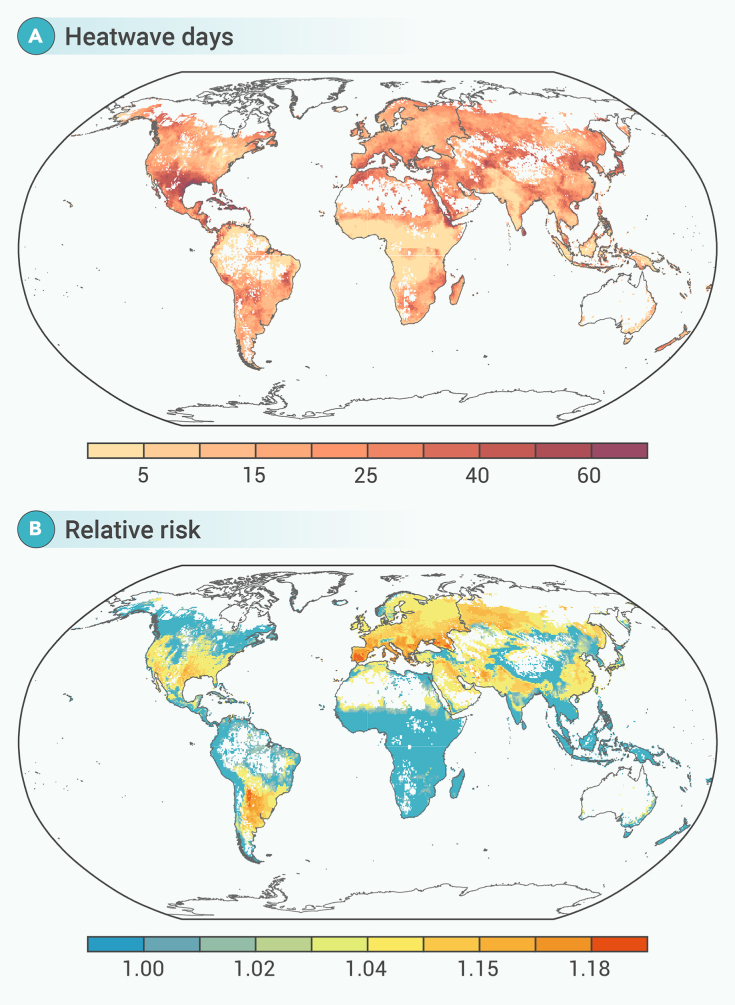
Heatwaves and associated mortality risk per global grid cell (0.5° × 0.5°) during the hottest months in 2023 (A) Number of heatwave days per global grid cell and (B) grid-specific relative risk of death (unitless).

In the third stage, we predicted heatwave-mortality associations (RR) in 2023 for each grid cell (0.5° × 0.5°) by applying the pooled mortality risks from past heatwaves, obtained through the meta-regression analysis, to the number of heatwave days in 2023, and other grid-level meta-predictors (GDP per capita, temperature metrics, climate classification, and region). Because of the data scarcity, we used the country-specific yearly mortality rates from 2000 to 2019 and interpolated these rates (using natural spline interpolation) to estimate the annual mortality rate for 2023. We then transformed this into grid cell-specific annual mortality counts using gridded population data, which were further converted to average daily mortality by dividing by the number of days in the calendar year. Heatwave-related excess deaths (ED) were calculated using the following formula:

$${ED}_{g}=\left({RR}_{g}-1\right)\times N_{g\;}\times D_{g}$$

where ${RR}_{g}$ is the grid cell-specific cumulative RR obtained from the grid cell-level heatwave-mortality risk predicted in the third stage (Figures 2C and 2D), and $N_{g\;}$ is the number of heatwave days per grid cell during the hottest months in 2023 (Figure 2A). $D_{g}$ is the daily average number of deaths per grid cell during the hottest months, which we estimated following our previous study.^16^ To estimate $D_{g}$ we first modeled the location-specific association between the ratio of deaths during the hottest months to year-round deaths, using yearly average temperature and temperature range as predictors in a linear regression. This model also accounted for indicators such as continents, climate zones, and GDP per capita. Next, we used the model outcome to estimate the grid-specific death ratio during the hottest months, using grid cell-specific yearly meta-predictors. Finally, *D*_*g*_ was calculated by multiplying the estimated grid cell-specific death ratio by the grid-level death count, which was derived from the annual mortality rate and population size per grid cell.

Uncertainty in estimating excess deaths was quantified by calculating the empirical confidence intervals (eCIs) using 500 Monte Carlo simulations. We calculated the number of excess deaths, excess death ratio (i.e., the ratio [%] of heatwave-related excess deaths to total deaths during the hottest months), and excess death rate (per million people) attributable to heatwave at global, continental and regional levels, based on the United Nations-defined regions. Only grid cells with at least one annual death during the study period were included in the analysis. These grids accounted for 98.5% of the global population.

To assess the contribution of human-induced climate change to heatwave-related mortality, we compared heatwave-related deaths under factual and counterfactual climate scenarios.^17^ We obtained the contribution rate (proportion) of human-induced climate change by dividing the difference in heatwave-related mortality estimates between the two scenarios by the estimate from the factual scenarios. This proportion was then multiplied by the heatwave-related death estimated by the third-stage model to quantify the deaths attributable to human-induced climate change. This approach helped to control the bias associated with differences between climate scenarios, ensuring more accurate estimate of the climate change attributable mortality.

To test the strength of our findings, we performed a series of sensitivity analyses by changing the maximum lag from 10 days to 14 and 21 days, and the number of degrees of freedom from 4 to 6 for both heatwave, lag dimension, and incorporating greenness (expressed as NDVI), and the proportion of the population aged 65 years and above (Table S4). We conducted additional sensitivity analyses using an alternative heatwave definition based on the 97.5th percentile with a duration of ≥2 days (Table S5), changing the metrics for heatwave definition to daily minimum and maximum temperatures (Table S6). Furthermore, we examined how variations in the number of climate models influence heatwave-related mortality estimates (Table S7).

All data analyses were done using R software, version 4.4.3. The dlnm package and the mixmeta packages were used to fit the distributed lag non-linear model and the meta-regression, respectively.^30^ This study was approved by the Monash University Human Research Ethics Committee (ID 24439).

### Role of the funding source

The funders of the study had no role in study design, data collection, data analysis, data interpretation, or writing of the report.

## Results

The global average temperature of the four hottest months in 2023 was 21°C, comprising an increase of 0.81°C compared with the average temperature during 2004–2010 (Figure S1). Many regions experienced heatwaves, with the highest median occurring in Central Asia (25 days), Southern Europe (25 days), Western Asia (25 days), and North Africa (24 days) (Table S8). From 2004 to 2023, the number of heatwave days showed an increasing trend, peaking in 2023 (Figure S2). The grid cell-level map of heatwave days further highlights variations within the regions (Figure 2A). Areas that experienced high number of heatwave days also had higher risk of death associated with heatwave (Figures 2A, 2B, and S3).

Globally, 178,486 excess deaths (95% eCI, 159,892−204,147) were attributed to heatwaves in 2023 (Table 1). Asia observed the highest heatwave-related mortality, accounting for 47.97% (85,611 deaths) of the global excess death, followed by Europe (37.23%, 66,443 deaths), the Americas (13.15%, 23,467deaths), Africa (1.61%, 2,881 deaths), and Oceania (0.05%, 83 deaths). Globally, the heatwave-related excess deaths accounted for 0.73% (95% eCI, 0.65–0.83%) of the total deaths during the hottest months, corresponding to 23 deaths per million people.

**Table 1:** Heatwave-related excess deaths (with 95% eCI) in 2023 and the contribution of human-induced climate change, globally and by continent

Heatwave-related mortality burden					Heatwave-related mortality burden attributable to human-induced climate change		
	Excess death (95% eCI)	Regional proportion (%)	Excess death ratio (%) (95% eCI)	Excess death rate per million people (95% eCI)	Excess deaths (95% eCI)	Excess death ratio (%) attributable to climate change (95% eCI)	Proportion of heatwave-related mortality attributable to human-induced climate change (%) (95% eCI)
Global	178,486 (159,892−204,147)	100.00	0.73 (0.65–0.83)	22.51 (20.16–25.74)	96,900 (73,089−125,266)	0.39 (0.30–0.51)	54.29 (45.71–61.36)
Africa	2,881 (1,566−5,881)	1.61	0.06 (0.03–0.13)	2.02 (1.1–4.12)	1,971 (115−4,982)	0.04 (0–0.11.00)	68.42 (7.37–84.71)
Northern Africa	2,063 (1,202−3,483)	1.16	0.32 (0.19–0.54)	7.93 (4.62–13.39)	1,444 (322−2,917)	0.23 (0.05–0.45)	69.98 (26.8–83.74)
Sub-Saharan Africa	818 (364−2,398)	0.46	0.02 (0.01–0.06)	0.70 (0.31–2.05)	527 (−207−2,065)	0.01 (−0.01−0.05)	64.48 (−56.87–86.12)
Americas	23,467 (20,630−28,014)	13.15	0.74 (0.65–0.88)	21.94 (19.29–26.19)	15,744 (12,322−20,468)	0.49 (0.39–0.64)	67.09 (59.73–73.06)
Latin America and the Caribbean	8,436 (7,139−11,255)	4.73	0.47 (0.4–0.63)	12.60 (10.66–16.81)	6,079 (4,529−8,872)	0.34 (0.25–0.49)	72.06 (63.44–78.83)
Northern America	15,031 (13,491−16,759)	8.42	1.08 (0.97–1.20)	37.56 (33.72–41.88)	9,666 (7,793−11,595)	0.69 (0.56–0.83)	64.31 (57.76–69.19)
Asia	85,611 (75,136−99,524)	47.97	0.65 (0.57–0.75)	18.47 (16.21–21.48)	54,636 (41,541−69,732)	0.41 (0.31–0.53)	63.82 (55.29–70.07)
Central Asia	1,551 (1,330−1,792)	0.87	0.98 (0.84–1.13)	22.77 (19.53–26.31)	1,088 (833−1,345)	0.69 (0.53–0.85)	70.14 (62.63–75.04)
Eastern Asia	56,488 (50,048−63,136)	31.65	1.15 (1.02–1.29)	35.98 (31.88–40.21)	3,8481 (30,910−45,888)	0.79 (0.63–0.94)	68.12 (61.76–72.68)
South-eastern Asia	1,416 (1,312−2,620)	0.79	0.08 (0.07–0.14)	2.09 (1.93–3.86)	1,068 (882−2,251)	0.06 (0.05–0.12)	75.41 (67.19–85.88)
Southern Asia	17,958 (15,889−22,088)	10.06	0.32 (0.28–0.39)	8.95 (7.91–11.00)	9,315 (6,220−13,618)	0.16 (0.11–0.24)	51.87 (39.14–61.65)
Western Asia	8,197 (6,556−9,88)	4.59	1.32 (1.06–1.59)	26.42 (21.13–31.87)	4,682 (2,697−6,631)	0.76 (0.43–1.07)	57.12 (41.14–67.06)
Europe	66,443 (62,502−70,562)	37.23	1.93 (1.82–2.05)	88.21 (82.98–93.68)	24,498 (19,116−29,963)	0.71 (0.56–0.87)	36.87 (30.58–42.46)
Eastern Europe	30,180 (28,249−32,151)	16.91	1.97 (1.84–2.10)	107.16 (100.3–114.16)	5,563 (2,749−8,385)	0.36 (0.18–0.55)	18.43 (9.73–26.08)
Northern Europe	3,614 (3,145−4,09)	2.02	0.90 (0.78–1.02)	32.87 (28.6–37.25)	874 (184−1,560)	0.22 (0.05–0.39)	24.19 (5.85–38.08)
Southern Europe	19,522 (18,737−20,367)	10.94	2.82 (2.71–2.94)	120.38 (115.54–125.5)	12,338 (11,435−13,274)	1.78 (1.65–1.92)	63.20 (61.03–65.17)
Western Europe	13,126 (12,371−13,948)	7.35	1.61 (1.52–1.72)	65.81 (62.02–69.92)	5,723 (4,747−6,745)	0.70 (0.58–0.83)	43.60 (38.37–48.36)
Oceania	83 (61–160)	0.05	0.06 (0.05–0.12)	1.81 (1.33–3.49)	44 (−5−121)	0.03 (0.00–0.09)	53.52 (−8.75−75.79)
Australia and New Zealand	80 (58–126)	0.04	0.08 (0.06–0.13)	2.30 (1.68–3.63)	41 (−6−88)	0.04 (−0.01−00.09)	51.63 (−9.60−70.07)
Other regions in Oceania^a^	3 (2.5–38)	0.00	0.01 (0.01–0.11)	0.29 (0.23–3.41)	3 (0.4–37)	0.01 (0.002–0.11)	99.09 (11.00–99.99)

% represents percentage or ratio. eCI, empirical confidence interval. The death ratio is calculated for the hottest months only. Regional groupings in this table are defined according to the UN Statistics Division regional groupings.

a Other regions in Oceania are defined as all areas in Oceania excluding Australia and New Zealand.

Heatwave-related mortality burden varied considerably across regions (Table 1). The highest mortality rate was observed in Southern Europe (120 deaths per million, 95% eCI, 116−126), Eastern Europe (107 deaths, 95% eCI, 100−114), and Western Europe (66 deaths, 95% eCI, 62−70), each exceeding three times the global average. North America, Eastern, and Western Asia also experienced considerably high excess death rates, while Sub-Saharan Africa, South-eastern Asia, and regions of Oceania obserevd the lowest. The excess mortality ratio showed a similar pattern, with slightly higher values in Southern (2.82%, 95% eCI, 2.71%–2.94%), Western (1.16%, 95% eCI, 1.52%–1.72%), and Eastern Europe (1.97%, 95% eCI, 1.84%–2.10%) (Table 1).

**Figure 3:**
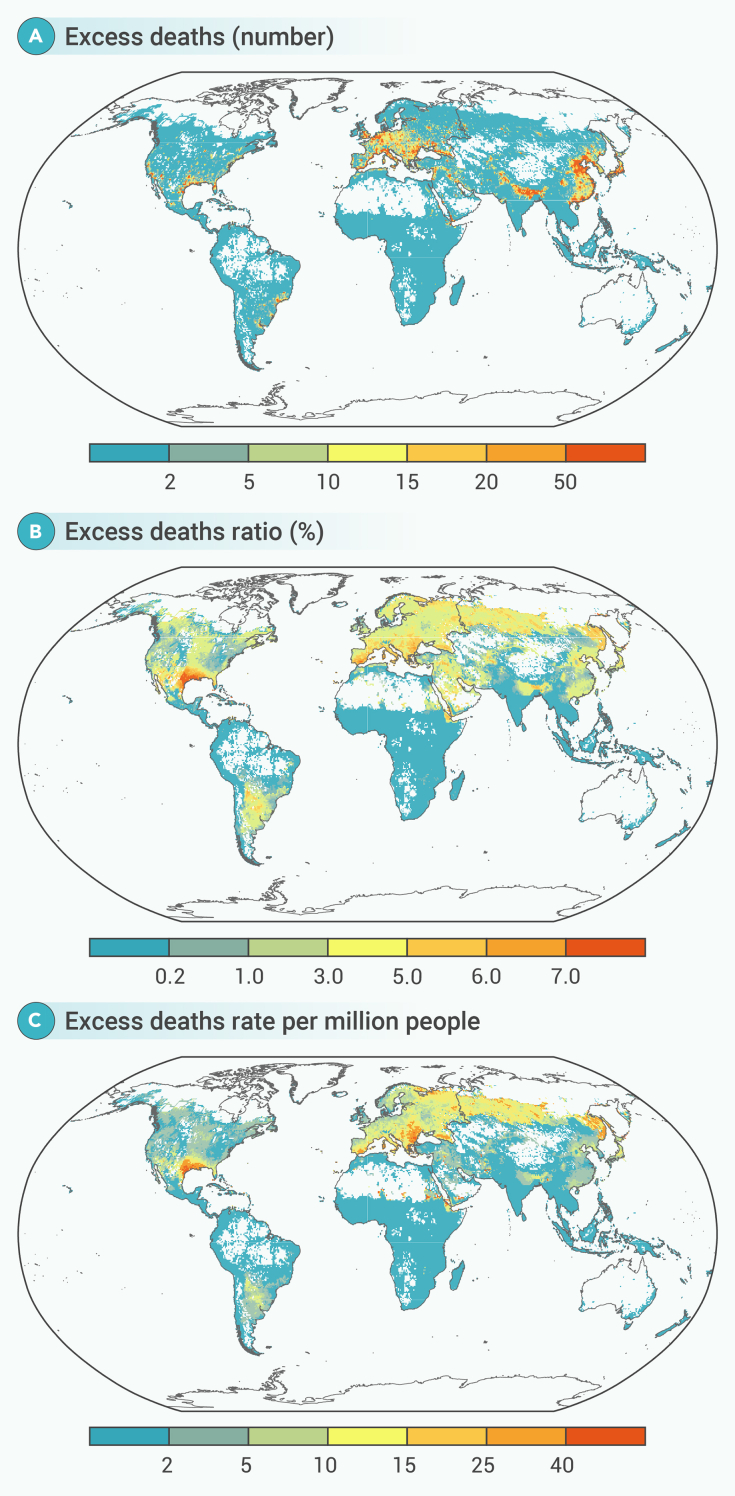
Grid cell-specific mortality burden associated with heatwave during the hottest months in 2023 (A) Fxcess deaths, (B) excess death ratio (%), and (C) excess death rate per million people. Our analysis focused on grid cells with a resolution of 0.5° × 0.5°, and only cells with at least one annual death event were included.

Figure 3 provides a detailed global overview of heatwave-related mortality, highlighting regional variations in the mortality burden. Notably, in Southern Europe, the grid-specific mortality ratio was greatly concentrated along the southern areas bordering the Mediterranean Sea. In North America, the excess death ratios were higher in parts of the western and southern United States. The distribution of grid cell-specific mortality rates follows a similar pattern (Figure 3C). Overall, grid cells with higher excess mortality burden were concentrated, largely in the subtropics and temperate zones of the Northern Hemisphere (Figures 3A–3C).

Human-induced climate change was responsible for 96,893 (95% eCI, 73,089–125,266) of all heatwave-related deaths globally, which represents 0.39% (95% eCI, 0.30%–0.51%) of all deaths during the hottest season. Overall, 54.29% of heatwave-related deaths were attributable to human-induced climate change (95% eCI, 45.71%**−**61.36%). However, this proportion varied substantially by regions, with the largest contributions (>60%) in South-eastern Asia, Latin America and the Caribbean, Central Asia, Eastern Asia, Southern Europe, Northern America, and Northern Africa (Table 1).

Sensitivity analyses show that our findings are robust. By testing different lag periods and degrees of freedom, we have confirmed that our results remain consistent (Table S4), reinforcing the validity of our conclusions. In a post-hoc analysis using the heatwave definition of the 97.5th percentile for ≥2 days, we found that the results were similar to the main analysis (Table S5). We found no significant difference in the mortality burden when comparing heatwaves defined by minimum temperatures and maximum temperatures with those defined by mean temperature. Notably, the estimated excess mortality burden associated with the 2023 heatwave remained unchanged when incorporating greenness and the proportion of the population aged 65 years and above as additional meta-predictors Table S6.

## Discussion

Our study estimated over 178,000 excess deaths globally due to heatwaves in 2023, equivalent to approximately 0.73% (95% eCI, 0.65%–0.83%) of all deaths during the hottest months. This translates to approximately 23 deaths per million people worldwide. Compared with previous estimates, such as the global average of 153,078 annual heatwave-related deaths during 2000–2019,^16^ our findings represent a marked increase, which can be partly attributable to the unprecedented severity and scale of the 2023 heatwave.^1^^,^^2^

We found that about 54.29% of all the heatwave-related deaths in 2023 were attributable to human-induced climate change. This coincides with previous attribution studies^17^ but reveals a broader impact likely due to our more extensive spatial coverage and heatwave-mortality modeling framework. Unlike earlier studies, which are often restricted to selected locations or cities, our analysis incorporated data from a diverse range of geographic settings and climate zones. This allowed us to identify regional disparities in the climate-related mortality burden more comprehensively. Although the degree of impact varied by location, the effect of human-induced climate change on heatwave mortality was evident across all study regions. These findings emphasize the growing influence of human-driven climate change in amplifying the health risks associated with heatwaves.

The adverse effects of heatwaves on human health arise from both direct and indirect mechanisms.^31^ During a heatwave, the body attempts to cool itself through sweating and vasodilation, but prolonged or excessive heat exposure can overwhelm these systems. Excessive sweating leads to dehydration, electrolyte imbalance, and hemoconcentration, which can exacerbate cardiovascular strain. Dehydration also increases sympathetic nervous system activation and heart rate, compounding risks, especially for vulnerable populations with pre-existing health conditions.^12^^,^^32^ Heatwaves can also disrupt critical public services, such as transportation, water supply systems, power grids, and healthcare delivery, further amplifying health risks during extreme events.^11^ These compounding factors make heatwaves a multifaceted public health emergency.

Despite the growing evidence of the deadly consequences of heatwaves, many countries remain unprepared to manage the health impacts of climate change. According to a recent WHO report, only half of the participating countries have a national health and climate change plan, and merely a quarter have successfully implemented such plan.^16^ Without decisive action, the mortality burden of heatwaves is likely to increase in the future.

Our findings indicate notably higher heatwave-related mortality during the summer of 2023, with nearly half of the heatwave-related excess deaths occurring in Asia, and Europe bearing the second highest burden. This finding coincides with earlier studies,^16^ although our estimates for Europe slightly exceed previous reports.^14^^,^^33^ This discrepancy may reflect a difference in methodology, data source, or spatial coverage. Previous studies in Europe primarily focused on selected cities in 35 countries, excluding the 2 largest countries on the continent. In contrast, our study quantifies heatwave-related deaths across all habitable grid cells in all European countries, not just in selected cities. This broader spatial coverage provides more detailed insights, capturing mortality patterns potentially missed by city-level analyses.

Our findings also showed significant geographical disparities within Europe. Southern and Western European regions had the highest excess mortality rates, exceeding the global average by more than three times. These findings are consistent with previous research highlighting that heat-related deaths in these regions are more than double the global average.^16^^,^^19^ Southern and Eastern European countries bordering the Mediterranean, such as Italy, Greece, Spain, and Portugal, reported high rates.^14^ These geographical patterns are largely attributable to climate factors as Europe exhibited record-breaking summer temperatures in 2023, particularly in the south. For example, temperatures surpassed 45°C in parts of Greece, eastern Spain, and southern Italy in July, prompting a surge in heat-related illnesses and deaths. Large parts of Italy and Spain issued heat warnings throughout most of July.^2^ Despite the implementation of national heat prevention plans since 2003, these findings underscore the ongoing challenge of mitigating heatwaves impact across Europe.

Beyond Europe, our study indicated wider geographical patterns in heatwave-related mortality. Grids with higher mortality rates were largely concentrated in the subtropics and temperate zones of the Northern Hemisphere. This coincides with previous research emphasizing the influence of climate zones on the geographical distribution of excess mortality. In 2023, the Northern Hemisphere saw a temperature increase of 1.54°C above the 20th century average compared with a 0.82°C increase in the Southern Hemisphere.^33^ The larger landmass in the Northern Hemisphere may contribute to the difference in temperature patterns. However, climate factors may not fully explain this disparity. Population acclimatization also plays a vital role. People living in regions that routinely experience high temperatures, such as tropical and equatorial areas, tend to have higher heat tolerance. Studies show that regions with higher temperatures, or near the equator, have higher minimum mortality temperatures compared with countries further from the equator.^27^^,^^28^ Moreover, frequent exposures to high temperatures have been associated with reduction in heat-related mortality risk,^34^ a trend also supported by a recent global study showing that the largest decline in heatwave-related mortality burden occurred in tropical regions.^16^ This suggests that adaptive capacity, both physiological and social, may shield the impacts of heatwaves in populations with more frequent exposure, whereas populations in cooler climates may remain more vulnerable despite shorter or less-intense heatwave events.^15^

Reducing vulnerability to heatwaves requires strong adaptation strategies. Strategies such as urban greening, increasing tree canopy coverage, and designing climate-resilient infrastructure can help lower temperatures in urban areas. Enhancing energy-efficient building design, improving ventilation, and insulation can protect individuals from indoor and workplace heat stress. Additionally, developing and expanding heat-health warning systems, conducting education campaigns, and improving healthcare system preparedness are important components of a coordinated response. Individual-level protection, community engagement, and intersectoral collaboration will be crucial in minimizing the health impact of heatwaves.^35^

It incorporated mortality and temperature data from a wide geographical area, encompassing 2013 locations across 67 countries/territories. This global scope ensures that the study’s findings are representative of diverse climates, socioeconomic conditions, and demographics, leading to more robust conclusions. The study utilizes well-established statistical models that account for the complex relationship between heatwaves and mortality, while addressing the influence of various confounding factors, allowing a more precise estimation of heatwave-related excess mortality. The analysis goes beyond national or regional levels by using high spatial resolution, providing valuable insights for local and regional decision-makers. By incorporating the latest data from 2023, the study provides timely and up-to-date scientific evidence on the global public health impact of heatwaves. The findings provide critical information to health authorities, supporting the development of effective heatwave protection strategies in the context of climate change.^5^

The study also has some limitations. Although we included key factors influencing geographic variations in the heatwave-mortality relationship,^16^^,^^18^ there could be other factors contributing to regional disparities. Due to the availability of daily mortality data for 2023, we assumed historical heatwave risks remained constant and used pre-pandemic epidemiological data to avoid the effect of COVID-19. Mortality rates for 2023 were interpolated from country-specific data spanning 2000–2019,^19^ minimizing pandemic influence but results should be interpreted carefully, given the pandemic’s widespread impact on health determinants and mortality since 2020. We used yearly GDP per capita and population data for grid-specific estimates, consistent with standard practices in the field,^16^^,^^19^ as such data are typically not available at a smaller temporal scale. Our analysis covers 67 countries/territories, but we have sparse historical mortality data from parts of Africa, Southern Asia, and Western Asia, which could potentially affect the accuracy in these regions. However, this is unlikely to substantially influence our results, as our model reduces uncertainty by borrowing information from similar locations. Future research should benefit from improved data collection in underrepresented regions and explore adaptation measures to enhance understanding. Due to data limitations, adaptation indicators such as access to air conditioning and healthcare resources were not included, and GDP per capita was used as a proxy.

## Conclusion

In summary, a substantially high global mortality burden was associated with the heatwaves in 2023, with the impact varying geographically. There was a considerable contribution of human-induced climate change. These emphasize the urgent need for intervention strategies that mitigate the impacts of climate change and protect public health during heatwave events.

## Resource availability

### Materials availability

This study did not generate new unique materials or reagents.

### Data and code availability

Mortality data used in our study are available from the local official authorities of each study country or territory under a data-sharing agreement. For information on accessing the data for each country or territory, investigators can refer to the MCC Collaborative Research Network participants, listed in the author or collaborator list. Observed temperature data were obtained from weather stations in each country and can be accessed by directly contacting the relevant agencies in the respective countries. Gridded temperature data were accessed from the publicly available ERA5 datasets,^21^ and the Detection and Attribution Model Intercomparison Project (DAMIP) (http://damip.lbl.gov/). No new data were created or analyzed in this study.

## Funding and acknowledgments

This study was supported by the 10.13039/501100000923Australian Research Council (DP210102076) and the Australian National Health and Medical Research Council (APP2000581). W.H. was supported by the 10.13039/501100004543China Scholarship Council (no. 202006380055). Z.Y. was supported by the Monash Graduate Scholarship and the Monash International Tuition Scholarship. S.L. was supported by an Emerging Leader Fellowship of the Australian National Health and Medical Research Council (no. APP2009866). Q.Z. was supported by the Program of Qilu Young Scholars of 10.13039/100009108Shandong University, Jinan, China. Y.G. was supported by the Fellowship (no. APP2008813) of the Australian National Health and Medical Research Council. R.X. was supported by the VicHealth Postdoctoral Fellowship 2022. S.T. was supported by the 10.13039/501100003399Science and Technology Commission of Shanghai Municipality (grant no. 18411951600), China. A.G. was supported by the EU’s Horizon 2020 project, Exhaustion (grant no. 820655), and Wellcome-funded project BREATHE (grant no. 308914/Z/23/Z). A.U. received funding from the Czech Ministry of Education Youth and Sport's programme ERC CZ (ID: LL2410). A.T. was supported by the JSPS KAKENHI (grant no. 24K13527). F.S. was supported by the 10.13039/501100000265Medical Research Council, United Kingdom (grant no. MR/R013349/1), the 10.13039/501100000270Natural Environment Research Council United Kingdom (grant no. NE/R009384/1), and the EU’s Horizon 2020 project, Exhaustion (grant no. 820655). V.H. was supported by a ‘Ramón y Cajal’ fellowship program of the Spanish Ministry of Science and Innovation (no. RYC2022-036948-I). Y.G. was supported by the Leader Fellowship (no. APP2008813) of the Australian National Health and Medical Research Council. The funders had no role in study design, data collection and analysis, decision to publish, or preparation of the manuscript.

## Author contributions

Y.G., A.G., and S.L. set up the collaborative network. Y.G. and S.L. conceptualized and designed the methodology. S.H. took the lead in the data analysis, results interpretation, and wrote the original draft of the manuscript. W.H. contributed to the methodology. All authors contributed to the data collection, revision, and editing of the manuscript. S.H., Y.G., and S.L. accessed and verified the data. All authors contributed to the manuscript and approved the final version.

## Declaration of interests

The authors declare no competing interests.

## Supplemental information

Document S1. Figures S1–S8 and Tables S1–S8

Document S2. Article plus supplemental information
