# Supplementary material for: Global excess deaths associated with heatwaves in 2023 and the contribution of human-induced climate change
Source: Innovation (Camb). 2025 Sep 4;6(10):101110. doi: 10.1016/j.xinn.2025.101110 (PMC7618246; doi:10.1016/j.xinn.2025.101110)
Supplement: Document S1. Figures S1–S8 and Tables S1–S8 [file mmc1.pdf]

The Innovation, Volume *6*

Supplemental Information

Global excess deaths associated with heatwaves in 2023 and the contribution of human-induced climate change

Samuel Hundessa, Wenzhong Huang, Rongbin Xu, Zhengyu Yang, Qi Zhao, Antonio Gasparrini, Ben Armstrong, Michelle L. Bell, Veronika Huber, Aleš Urban, Micheline Coelho, Francesco Sera, Shilu Tong, Dominic Royé, Jan Kyselý, Francesca de'Donato, Malcolm Mistry, Aurelio Tobias, Carmen Íñiguez, Martina S. Ragettli, Simon Hales, Souzana Achilleos, Jochem Klompmaker, Shanshan Li, Yuming Guo, and Multi-Country Multi-City Collaborative Research Network

Supplemental Information

Table of Contents

**Materials and Methods**	Description of the climate scenarios datasets used for the study.
**Table S1.**	Summary of location-specific deaths and temperatures by country/territory and period.
**Table S2.**	Description of the climate models used for climate change attribution.
**Table S3.**	Second-stage random-effects meta-regression models examining heatwave definitions at the 95th percentile threshold for >2 days, including Cochran's Q test p-values and I^2 for residual heterogeneity.
**Table S4.**	Sensitivity analysis on the degrees of freedom (df) for exposure-response and different lag dimensions.
**Table S5.**	Heatwave-related mortality based on periods of ≥2 days with daily mean temperatures above 97.5th percentiles for each location.
**Table S6.**	Comparison of Heatwave-related mortality estimates based on different heatwave definitions, adjusted for green space and population proportion.
**Table S7.**	Heatwave-related mortality burden estimated under the Factual scenario using varying numbers of climate models.
**Table S8.**	Global and regional average temperature and number of heatwave days during the hottest months in 2023.
**Figure S1.**	Annual average temperature of the hottest months during 2004-2023 and annual changes compared to the 2004-2010 average.
**Figure S2.**	Trends in the number of heatwave days (median) during 2004-2023.
**Figure S3.**	Regional estimates of heatwave mortality risk (RR) with 95% empirical confidence intervals.

Materials and Methods

Description of the climate scenarios datasets used for the study.

We utilized two climate scenarios: one representing historical (factual) climate conditions and the other a counterfactual scenario approximating a hypothetical world without anthropogenic climate change. The temperature data for these scenarios (near-surface air temperature) were extracted from the Detection and Attribution Model Intercomparison Project (DAMIP) climate database[1], which is part of CMIP6. DAMIP is specifically designed to assess the individual contributions of various external factors to past and future changes in global and regional climates. This study incorporated ensemble member simulations from four general circulation models (CanESM5, IPSL-CM6A-LR, MIROC6, and NorESM2-LM) within CMIP6, drawn from two different experiments for which relevant data were available at the time of analysis. Details about the models and selected simulations can be found in Table S2. For the factual scenario, we used historical climate simulations (‘hist’) of mean daily temperatures available up to 2014, combined with simulations of ssp2rcp45 for the subsequent years until 2023[2]. These simulations account for all types of natural and anthropogenic forcing, effectively mimicking the actual historical climate[1]. In contrast, the counterfactual climate data were derived from the ‘hist-nat’ experiment, which considers only natural forcing. This counterfactual dataset approximates a hypothetical climate devoid of human influences since the early twentieth century, allowing for a clear distinction between natural and anthropogenic climate change. Daily mean temperature series (near-surface air temperature—tas) were extracted from globally gridded datasets and bias-corrected using ERA5 data. In summary, the ERA5 temperature series was employed to bias-correct the factual scenario, and the same correction factors were applied to the modelled series of the counterfactual scenario.

Table S1. Summary of location-specific deaths and temperatures by country/territory and period.

Country/territory	Locations	Period	All-cause mortality (yearly average)	Temperature, annual average (ºC)*
**Argentina**	3	2005–2015	18767	23·7 (23·6, 23·9)
**Australia**	341	2009–2019	46785	21·8 (12·8, 32·5)
**Bangladesh**	1	2005–2016	124	26·8 (26·.8, 26·8)
**Brazil**	494	1996–2019	350094	24·5 (18·9, 28·9)
**Bulgaria**	5	2000–2019	8034	21·6 (19·5, 22·8)
**Burkina Faso**	3	1998–2015	880	29·3 (29·2, 29·4)
**Canada**	288	1986–2015	77431	15·1 (-0·2, 20·7)
**Chile**	15	2016–2019	33099	14·0 (7·1, 17·4)
**China**	15	1996–2015	124360	24·5 (17·6, 28·0)
**Colombia**	5	1998–2013	20348	23·8 (14·0, 28·7)
**Costa Rica**	1	2000–2017	580	22·7 (22·7, 22·7)
**Cyprus**	5	2004–2019	1534	26·1 (24·4, 27·6)
**Czech Republic**	4	1994–2019	10313	17·5 (16·6, 18·5)
**Ecuador**	2	2014–2018	7485	21·1 (15·5, 26·7)
**Estonia**	5	1997–2019	2414	15·1 (14·7, 15·7)
**Ethiopia**	4	2006–2015	437	18·3 (17·0, 19·5)
**Finland**	1	1994–2014	2356	15·1 (15·1, 15·1)
**France**	20	2000–2017	35938	18·8 (16·2, 22·8)
**French Guiana**	1	2000–2015	154	27·0 (27·0, 27·0)
**Germany**	15	1993–2019	49871	17·3 (16·3, 18.5)
**Ghana**	1	1994–2014	310	27·9 (27·9, 27·9)
**Greece**	1	2001–2010	9418	26·7 (26·7, 26·7)
**Guadeloupe**	1	2000–2015	568	27·7 (27·7, 27·7)
**Guatemala**	1	2009–2016	2639	20·3 (20·3, 20·3)
**Iceland**	1	2000–2018	423	10·0 (10·0, 10·0)
**India**	1	2009–2016	149	30·2 (30·2, 30·2)
**Iran**	2	2002–2015	26865	26·4 (25·7, 27·1)
**Ireland**	6	1984–2007	13494	13·6 (13·1, 14·0)
**Israel**	4	1985–2019	5130	25·4 (23·6, 26·7)
**Italy**	18	2006–2015	28083	23·1 (20·9, 24·9)
**Ivory Coast**	1	2009–2016	142	25·2 (25·2, 25·2)
**Japan**	47	1973–2019	293752	23·1 (18·1, 27·1)
**Kenya**	2	2003–2015	263	21·7 (19·6, 23·8)
**Kuwait**	1	2000–2016	1336	37·3 (37·3, 37·3)
**Malawi**	1	2003–2016	90	23·7 (23·7, 23·7)
**Malta**	1	1995–2019	963	24·5 (24·5, 24·5)
**Martinique**	1	2000–2015	435	27·9 (27·9, 27·9)
**Mexico**	11	1998–2019	59717	21·9 (14·5, 28·2)
**Moldova**	4	2001–2010	2354	20·6 (20·1, 21·1)
**Mozambique**	1	2010–2015	222	26·4 (26·4, 26·4)
**Netherland**	5	1995–2016	6562	16·3 (15·9, 16·7)
**New Zealand**	66	2000–2018	9170	14·5 (9·7, 17·9)
**Nigeria**	1	2011–2014	970	27·8 (27·8, 27·8)
**Norway**	1	1973–2018	1718	13·4 (11·4, 14·4)

**Panama**	1	2013–2016	970	28·3 (22·3, 29·3)
**Paraguay**	1	2004–2019	937	27·2 (25·2, 29·2)
**Peru**	18	2008–2014	29193	18·9 (5·5, 27·2)
**Philippines**	13	2006–2019	30700	28·8 (28·4, 29·5)
**Portugal**	6	1980–2018	14958	20·7 (18·1, 22·3)
**Puerto Rico**	1	2009–2016	1100	27·8 (27·8, 27·8)
**Reunion**	1	2000–2015	292	26·2 (26·2, 26·2)
**Romania**	8	1994–2016	13090	20·3 (17·4, 21·7)
**Senegal**	3	1990–2016	194	27·4 (26·4, 28·5)
**Serbia**	1	1995–2019	6460	21·9 (20·1, 23.9)
**South Africa**	55	1997–2013	158685	22·1 (14·6, 27·6)
**South Korea**	36	1997–2018	44553	22·5 (19·0, 24·0)
**Spain**	52	1990–2014	37643	21·4 (16·7, 26·0)
**Sweden**	3	1990–2016	8377	15·9 (15·8, 15·9)
**Switzerland**	8	1969–2018	4311	17·3 (15·3, 19·7)
**Taiwan**	6	2000–2018	29701	24·3 (21·5, 26·0)
**Tanzania**	3	1994–2014	567	25·4 (22·3, 27·3)
**Thailand**	62	1999–2008	62582	28·5 (26·7, 29·8)
**Gambia**	1	1990–2015	84	27·8 (23·8, 27·9)
**UK**	112	1990–2019	69241	15·3 (13·9, 16·7)
**USA**	211	1973–2006	354003	22·2 (13·4, 32·4)
**Uruguay**	1	2012–2016	9116	24·0 (24·0, 24·0)
**Vietnam**	3	2004–2013	9568	28.6 (27·7, 29·4)

*Average (range)

Table S2. Description of the climate models used for human-induced climate change attribution.

Climate Model	Member (variant label)	Citation
**CanESM5**	r1i1p1f1	Swart, N. C. et al. The Canadian Earth System Model version 5 (CanESM5.0.3). Geosci. Model Dev. Discuss. (2019) doi:10.5194/gmd-2019-177
**MIROC6**	r1i1p1f1	Tatebe, H. et al. Description and basic evaluation of simulated mean state, internal variability, and climate sensitivity in MIROC6. Geosci. Model Dev. (2019) doi:10.5194/gmd-12-2727-2019
**IPSL-CM6A-LR**	r1i1p1f1	Boucher, O., Servonnat, J., Albright, A. L., Aumont, O. & Balkanski, Y. Presentation and evaluation of the IPSL-CM6A-LR climate model. J. Adv. Model. Earth Syst. (2020) doi:10.1029/2019MS002010.
**NorESM2-LM**	r1i1p1f1	Seland, Ø. et al. The Norwegian Earth System Model, NorESM2 – Evaluation of the CMIP6 DECK and historical simulations. Geosci. Model Dev. Discuss. (2020) doi:10.5194/gmd-2019-378.

Daily mean temperature series (near-surface air temperature—tas) simulated by these models for both factual and counterfactual scenarios were extracted from the Detection and Attribution Model Intercomparison Project (DAMIP) (http://damip.lbl.gov/)

Table S3. Second-stage random-effects meta-regression models examining heatwave definitions at the 95th percentile threshold for ≥2 days, including Cochran's Q test p-values and I^2 for residual heterogeneity.

Model	Predictor	I^2 (%)	*p-value* for Q test
**Intercept only**	No predictor	34.3	<0.0001
**Full Model**	Annual average Temperature		
	Temperature range		
	Climate classifications	20.7%	<0.0001
	GDP per capita		
	Period		
	Regions		
	Annual average temperature: Climate classifications		
	Temperature range: Annual average temperature		
**Model with Relative Humidity**	Relative Humidity	21.0%	<0.0001
	Annual average Temperature		
	Temperature range		
	Climate classifications		
	GDP per capita		
	Period		
	Regions		
	Annual average temperature: Climate classifications		
	Temperature range: Annual average temperature		

I^2 (%) indicates the percentage of residual heterogeneity. p-value for Q test refers to Cochran's Q test for heterogeneity.

Table S4. Sensitivity analysis on the degrees of freedom (df) for exposure-response and different lag dimensions.

	Excess death ratio (%) (95% eCI)	Excess death rate per million people (95% eCI)
**Main model**	0·73 (0·65–0·83)	23 (20–26)
**Df for lag response: 6**	0·72 (0·65–0·88)	21 (17–25)
**The lag period of 14**	0·74 (0·71–0·83)	22 (16–26)
**A lag period of 21**	0·75 (0·63–0·79)	24 (19–37)
**Using heatwave definition of 97·5th percentile and ≥ 2days**	0·75 (0·66–0·88)	21 (18–24)
**Adjusting for both greenness (NDVI) and the proportion of the population above 65 years**	0·73 (0·52–0·99)	23 (16–31)
**Using minimum temperature for the heatwave definition**	0·75 (0·48–1·11)	23 (15–36)
**Using maximum temperature for the heatwave definition**	0·74 (0·68–0·81)	23 (21–25)

eCI=empirical confidence interval

Table S5. Heatwave-related mortality based on periods of ≥2 days with daily mean temperatures above 97.5th percentiles for each location.

	**Number of excess deaths (95% eCI)**	**Excess death ratio (%) (95% eCI)**	**Excess death rate per million people (95% eCI)**
**Global**	165,894 (145,773–193,431)	0·75 (0·66–0·88)	20·92 (18·38–24·39)
**Africa**	2,135 (924–5,306)	0·03 (0·01–0·08)	1·50 (0·65–3·72)
**Northern Africa**	1,543 (804–3,133)	0·17 (0·09–0·34)	5·93 (3·09–12·05)
**Sub-Saharan Africa**	592 (119–2,172)	0·01 (0·00–0·04)	0·51 (0·10–1·86)
**Americas**	27,878 (2,4497–33,152)	0·58 (0·51–0·69)	26·06 (22·9–30·99)
**Latin America and the Caribbean**	8,782 (7,239–12,026)	0·33 (0·27–0·45)	13·12 (10·81–17·96)
**Northern America**	19,096 (17,259–21,126)	0·91 (0·82–1·01)	47·72 (43·13–52·80)
**Asia**	70,316 (58,722–85,331)	0·37 (0·31–0·44)	15·17 (12·67–18·41)
**Central Asia**	1,087 (874–1,313)	0·46 (0·37–0·55)	15·96 (12·83–19·28)
**Eastern Asia**	45,255 (37,937–53,051)	0·63 (0·53–0·74)	28·82 (24·16–33·79)
**South-eastern Asia**	550 (496–1,565)	0·02 (0·02–0·06)	0·81 (0·73–2·31)
**Southern Asia**	17,456 (14,970–21,729)	0·21 (0·18–0·26)	8·70 (7·46–10·82)
**Western Asia**	5,968 (4,445–7,673)	0·64 (0·48–0·82)	19·24 (14·33–24·73)
**Europe**	65,508 (61,592–69,537)	1·27 (1·2–1·35)	86·97 (81·77–92·32)
**Eastern Europe**	28,128 (26,240–30,055)	1·24 (1·15–1·32)	99·87 (93·17–106·72)
**Northern Europe**	3,150 (2,765–3,547)	0·52 (0·46–0·59)	28·65 (25·14–32·26)
**Southern Europe**	23,179 (22,153–24,233)	2·23 (2·13–2·33)	142·93 (136·6–149·43)
**Western Europe**	11,051 (10,434–11,702)	0·91 (0·86–0·96)	55·4 (52·31–58·66)
**Oceania**	57 (43–100)	0·03 (0·02–0·05)	1·24 (0·93–2·18)
**Australia and New Zealand**	55 (41–85)	0·04 (0·03–0·06)	1·57 (1·18–2·45)
**Other regions in Oceania**	2 (1.6–15)	0·002 (0·001–0·034)	0·19 (0·15–1·34)

eCI = empirical Confidence Interval. Note: Regional groupings in this table are defined according to the UN Statistics Division regional groupings. *Other regions in Oceania are defined as all areas in Oceania excluding Australia and New Zealand. Only grids with at least one annual death were included.

Table S6. Comparison of Heatwave-related mortality estimates based on different heatwave definitions, adjusted for green space and population proportion.

	**Excess death (95% eCI)**	***P*-value for difference**
Temperatures used for heatwave definition<br>Daily minimum temperature	184,363 (119,250–274,216)	0.234
Daily maximum temperature	181,591 (166,223–198,341)	0.523
Daily mean temperature	178, 486 (159, 892–204, 147)	Reference
Adjusting for both greenness (NDVI) and the proportion of the population above 65 years	179,428 (131,637–252,983)	0.537

eCI: empirical Confidence Interval, p-value for the difference is from the t-test comparing heatwave-related mortality estimates derived from heatwave definitions based on daily minimum and maximum temperatures and including additional meta-predictors (greenness defined as NDVI) and the proportion of the population above 65 years compared to the mean temperature (main results).

Table S7. Heatwave-related mortality burden estimated under the Factual scenario using varying numbers of climate models.

	**Heatwave-related mortality (95% eCI)**		
	**4 GCMs**	**3 GCMs**	**2 GCMs**
**Global**	178072 (160232-203765)	182696 (165735-208474)	180970 (165021-206848)
**Americas**	25385 (22853-29763)	20492 (18371-24658)	20704 (18706-24736)
**Europe**	67778 (63834-71902)	79002 (74465-83650)	82290 (78219-86508)
**Africa**	4777 (3495-8364)	1523 (598-4761)	1620 (618-5086)
**Asia**	79842 (69786-93364)	81555 (72218-95153)	76241 (67399-90279)
**Oceania**	289 (68-367)	124 (86-246)	115 (81-234)

GCM = Global Climate Model; eCI: empirical Confidence Interval

Table S8. Global and regional average temperature and number of heatwave days during the hottest months in 2023.

	Annual mean temperature (ºC) (±SD)	Median heatwave days (IQR)
**Global**	21 ±8	18 (11, 25)
**Australia and New Zealand**	19 ±5	11 (7, 16)
**Central Asia**	21 ±8	25 (21, 30)
**Eastern Asia**	19 ±8	22 (15, 29)
**Eastern Europe**	15 ±6	19 (15, 23)
**Latin America and the Caribbean**	23 ±6	17 (11, 26)
**Northern Africa**	29 ±5	24 (17, 32)
**Northern America**	17 ±7	18 (12, 25)
**Northern Europe**	13 ±5	16 (13, 20)
**Other regions in Oceania**	25 ±3	9 (4, 17)
**South-eastern Asia**	26 ±2	14 (8, 22)
**Southern Asia**	27 ±8	17 8, 25)
**Southern Europe**	21 ±5	25 (22, 31)
**Sub-Saharan Africa**	26 ±4	6 (0, 17)
**Western Asia**	28 ±8	25 (19, 30)
**Western Europe**	18 ±4	19 (16, 23)

Note: Regional groupings in this table are defined according to the UN Statistics Division regional groupings. *Other regions in Oceania are defined as all areas in Oceania excluding Australia and New Zealand. Only grids with at least one annual death were included. SD= Standard Deviation, IQR= Interquartile Range

Supplemental Figures

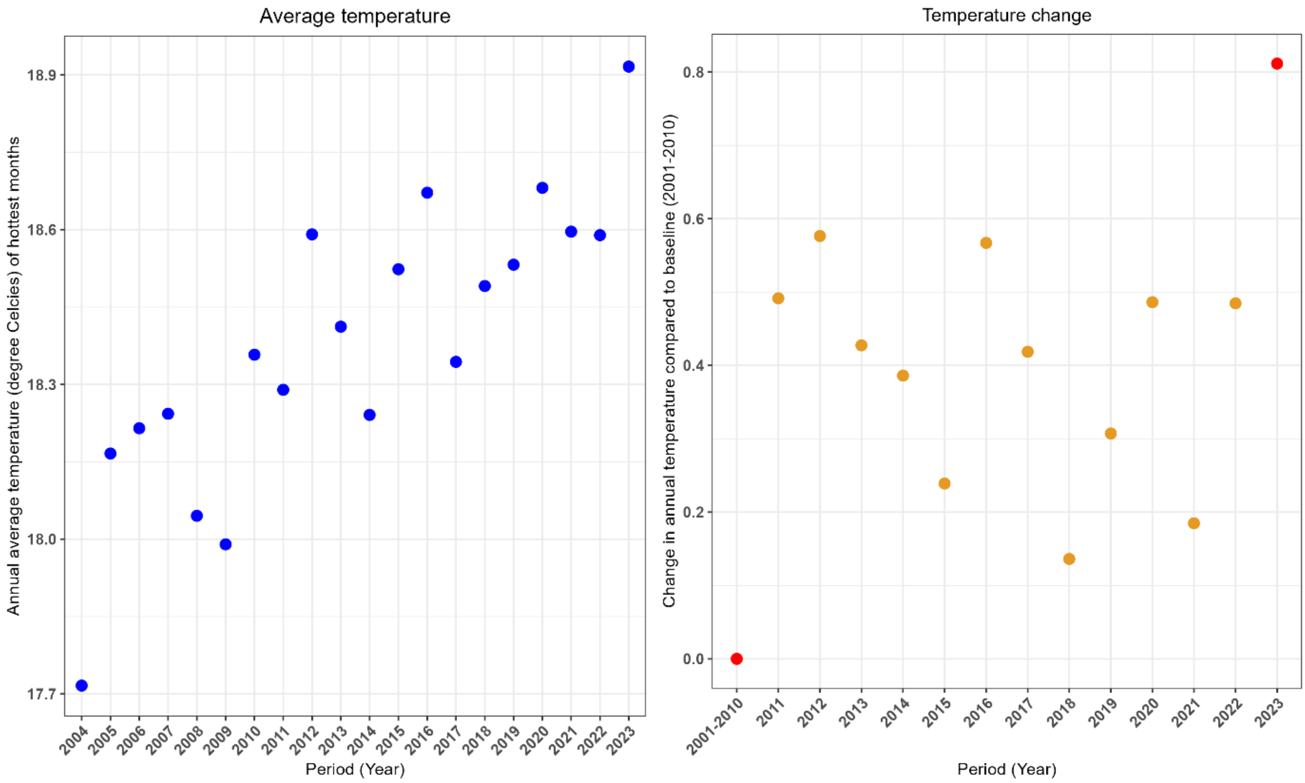

Figure S1. Average temperature of the hottest months during 2004-2023 (left) and annual changes compared to the 2004-2010 average (right). The red dot at the top right corner indicates the change in 2023.

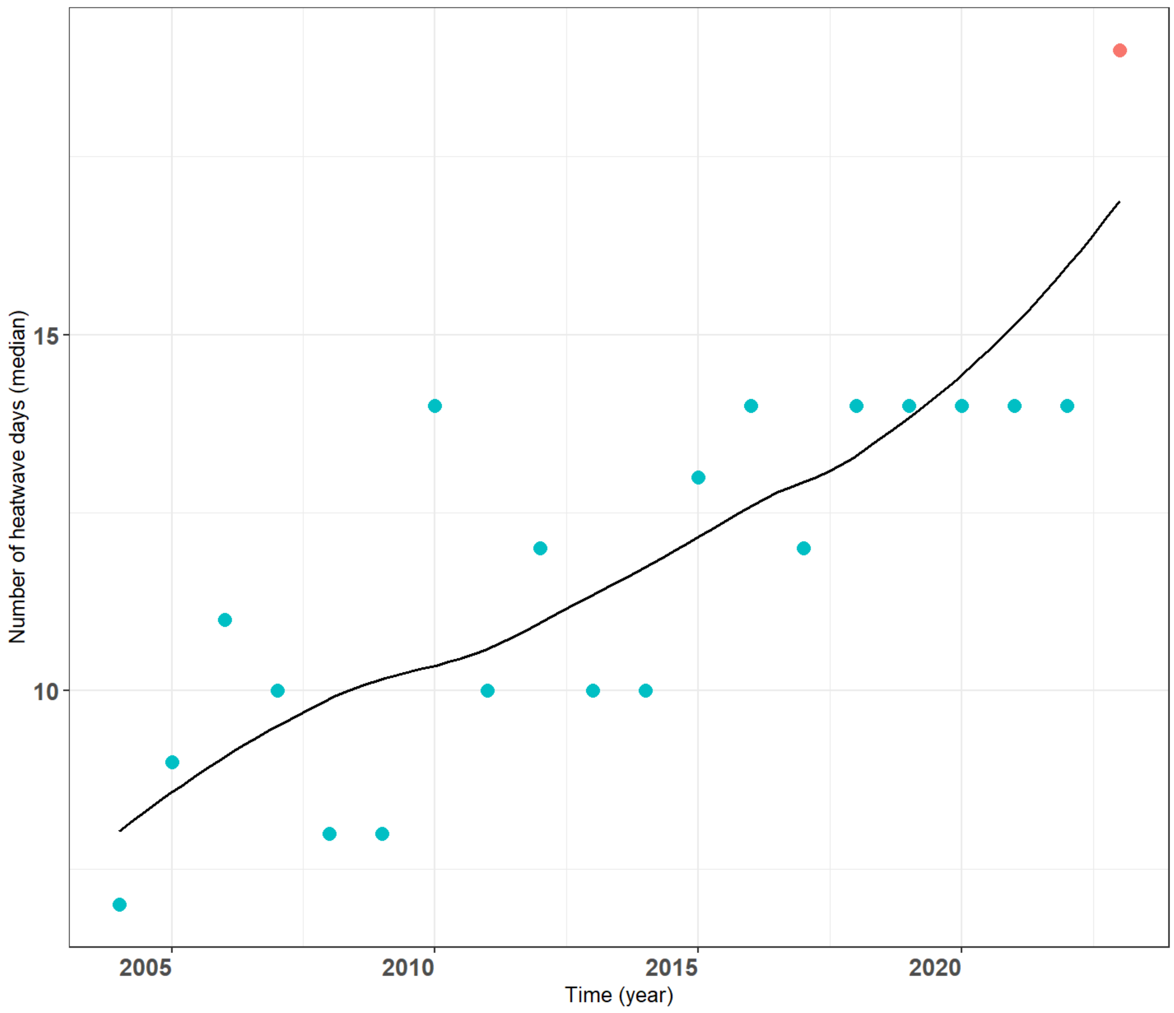

Figure S2. Trends in the number of heatwave days (median) during 2004-2023. The blue dots indicate the median number of heatwave days per year from 2004 to 2022, while the straight line shows the linear trend. The red dot indicates the median number of heatwave days in 2023.

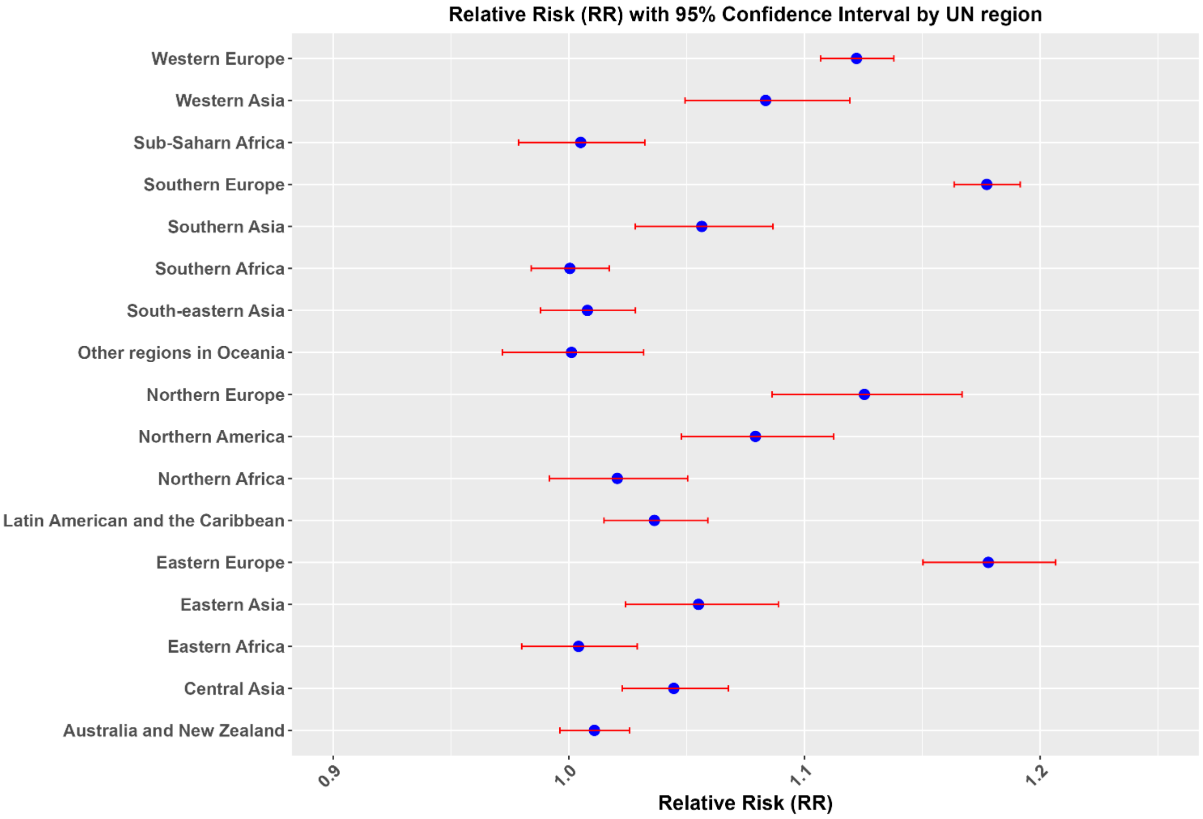

Figure S3. Regional estimates of heatwave mortality risk (RR) with 95% empirical confidence intervals. Regional groupings in this table are defined according to the UN Statistics Division regional groupings. *Other regions in Oceania are defined as all areas in Oceania excluding Australia and New Zealand. Only grids with at least one annual death were included.

[25]Multi-Country Multi-City Collaborative Research Network

Jouni Jaakkola,[25,26] Ana Maria Vicedo-Cabrera,[27,28,29] Rosana Abrutzky,[30] Paulo Saldiva,[31] Eric Lavigne,[32,33,34] Patricia Correa,[35,36], Nicolás Ortega,[37] Haidong Kan,[38] Samuel Osorio,[39] Hans Orru,[40] Ene Indermitte,[40] Jouni Jaakkola,[25,26] Niilo Ryti,[25,26] Mathilde Pascal,[41] Alexandra Schneider,[42] Veronika Huber,[6,43] Klea Katsouyanni,[44,45] Antonis Analitis,[44] Hanne Carlsen,[46] Fatemeh Mayvaneh,[47] Hematollah Roradeh[47], Patrick Goodman[48], Ariana Zeka[49], Raanan Raz,[50] Paola Michelozzi,[16] Masahiro Hashizume,[51] Yoonhee Kim,[52] Barrak Alahmad,[53] John Paul Cauchy,[54] Magali Diaz,[55] Eunice Arellano,[55] Ala Overcenco,[56] Shilpa Rao,[57] Gabriel Carrasco,[58] Xerxes Seposo,[59,60] Paul Chua,[51] Susana da Silva,[61] Joana Madureira,[62,63,64] Iulian-Horia Holobaca,[65] Ivana Cvijanovic,[66] Noah Scovronick,[67] Fiorella Acquaotta,[68] Ho Kim,[69] Whanhee Lee,[70,71] Bertil Forsberg,[72] Yue Guo,[73,74,75] Shih-Chun Pan,[74] Yasushi Honda,[76] Valentina Colistro,[77] Antonella Zanobetti,[78] Joel Schwartz,[78] Tran Dang,[79,80] Do Dung,[81] Paulo Sadiva,[9] Leon Guo[82]

Affiliations:

[25]Center for Environmental and Respiratory Health Research (CERH), University of Oulu, Oulu, Finland, [26]Medical Research Center Oulu (MRC Oulu), Oulu University Hospital and University of Oulu, Oulu, Finland, [27]Institute of Social and Preventive Medicine, University of Bern, Bern, Switzerland, [28]Oeschger Center for Climate Change Research, University of Bern, Bern, Switzerland, [29]Department of Public Health Environments and Society, London School of Hygiene and Tropical Medicine, London, United Kingdom, [30]Universidad de Buenos Aires, Facultad de Ciencias Sociales, Instituto de Investigaciones Gino Germani, [31]INSPER, São Paulo, Brazil, [32]School of Epidemiology & Public Health, Faculty of Medicine, University of Ottawa, Ottawa, Canada, [33]School of Epidemiology and Public Health, University of Ottawa, [34]Air Health Science Division, Heatlh Canada, Ottawa, Canada, [35]Department of Public Health, Universidad de los Andes, Santiago, Chile, [36]School of Medicine, University of the Andes (Chile), [37]Centro Interdisciplinario de Cambio Global, Pontificia, Universidad Católica de Chile, Santiago, Chile, [38]Department of Environmental Health, School of Public Health, Fudan University, Shanghai, China, [39]Department of Environmental Health, University of São Paulo, São Paulo, Brazil, [40]Department of Family Medicine and Public Health, University of Tartu, Tartu, Estonia, [41]Santé Publique France, Department of Environmental and Occupational Health, French National Public Health Agency, Saint Maurice, France, [42]Institute of

Epidemiology, Helmholtz Zentrum München – German Research Center for Environmental Health (GmbH), Neuherberg, Germany, [43]Department of Physical, Chemical and Natural Systems, Universidad Pablo de Olavide, Sevilla, Spain, [44]Department of Hygiene, Epidemiology and Medical Statistics, National and Kapodistrian University of Athens, Greece, [45]Environmental Research Group, School of Public Health, Imperial College, London, UK, [46]School of Public Health and Community Medicine, University of Gothenburg, Gothenburg, Sweden, [47]Geography and Urban Planning Department, University of Mazandaran, Babolsar, Iran, [48]Technological University Dublin, Ireland, [49]UK Health Security Agency, London, UK, [50]Braun School of Public Health and Community Medicine, The Hebrew University of Jerusalem, Israel, [51]Department of Global Health Policy, Graduate School of Medicine, The University of Tokyo, Tokyo, Japan, [52]Department of Global Environmental Health, Graduate School of Medicine, University of Tokyo, Tokyo, Japan, [53]Department of Environmental Health, Harvard T.H. Chan School of Public Health, Harvard University, Boston, MA, USA, [54](No affiliation data provided), [55]Department of Environmental Health, National Institute of Public Health, Cuernavaca, Morelos, Mexico, [56]National Agency for Public Health of the Ministry of Health, Labour and Social Protection of the Republic of Moldova, [57]Norwegian institute of Public Health, Oslo, Norway, [58]Institute of Tropical Medicine "Alexander von Humboldt", Universidad Peruana Cayetano Heredia, Lima, Peru, [59]Department of Hygiene, Graduate School of Medicine, Hokkaido University, Sapporo, Japan, [60]School of Tropical Medicine and Global Health, Nagasaki University, Nagasaki, Japan, [61]Department of Epidemiology, Instituto Nacional de Saúde Dr. Ricardo Jorge, Lisbon, Portugal, [62]Department of Environmental Health, Instituto Nacional de Saúde Dr. Ricardo Jorge, Porto, Portugal, [63]EPIUnit - Instituto de Saúde Pública, Universidade do Porto, Porto, Portugal, [64]Laboratório para a Investigação Integrativa e Translacional em Saúde Populacional (ITR), Porto, Portugal, [65]Faculty of Geography, Babes-Bolay University, Cluj-Napoca, Romania, [66]Barcelona Institute for Global Health, Barcelona, Spain, [67]Department of Environmental Health. Rollins School of Public Health, Emory University, Atlanta, USA, [68]Department of Earth Sciences, University of Torino, Italy, [69]Graduate School of Public Health, Seoul National University, Seoul, South Korea, [70]School of Biomedical Convergence Engineering, College of Information and Biomedical Engineering, Pusan National University, Yangsan, South Korea, [71]Institute of Ewha-SCL for Environmental Health (IESEH), [72]Department of Public Health and Clinical Medicine, Umeå University, Sweden, [73]Environmental and Occupational Medicine, National Taiwan University (NTU) College of Medicine and NTU Hospital, Taipei, Taiwan, [74]National Institute of Environmental Health Science, National Health Research Institutes, Zhunan,

Taiwan, [75]Graduate Institute of Environmental and Occupational Health Sciences, NTU College of Public Health, Taipei, Taiwan, [76]Center for Climate Change Adaptation, National Institute for Environmental Studies, Tsukuba, Japan, [77]Department of Quantitative Methods, School of Medicine, University of the Republic, Montevideo, Uruguay, [78]Department of Environmental Health, Harvard T.H. Chan School of Public Health, Boston, MA, USA, [79]Institute of Research and Development, Duy Tan University, Da Nang, Vietnam, [80]Department of Environmetal Health, Faculty of Public Health, Department of Environmetal Health, University of Medicine and Pharmacy at Ho Chi Minh City, Ho Chi Minh City, Vietnam, [81]Department of Environmetal Health, Faculty of Public Health, University of Medicine and Pharmacy at Ho Chi Minh City, Ho Chi Minh City, VietNam, [82]Institute of Environmental and Occupational Health Science, National Taiwan University.

References

1. Gillett NP, Shiogama H, Funke B, et al. The detection and attribution model intercomparison project (DAMIP v1. 0) contribution to CMIP6. *Geoscientific Model Development*. 2016;9:3685-3697.
2. Vicedo-Cabrera AM, Scovronick N, Sera F, et al. The burden of heat-related mortality attributable to recent human-induced climate change. *Nat Clim Chang*. 2021;11:492-500.
